# Comparative genomics of type VI secretion systems in strains of *Pantoea ananatis* from different environments

**DOI:** 10.1186/1471-2164-15-163

**Published:** 2014-02-26

**Authors:** Divine Yufetar Shyntum, Stephanus Nicolaas Venter, Lucy Novungayo Moleleki, Ian Toth, Teresa Ann Coutinho

**Affiliations:** 1 Department of Microbiology and Plant Pathology, Forestry and Agricultural Biotechnology Institute (FABI), University of Pretoria, Pretoria, South Africa; 2 James Hutton Research Institute, Invergowrie, Dundee DD2 5DA, Scotland, UK

**Keywords:** Type VI secretion system, T6SS, *Pantoea ananatis*, Phytopathogen

## Abstract

**Background:**

The Type VI secretion system (T6SS) has been identified in several different bacteria, including the plant pathogen*Pantoea ananatis*. Previous *in silico* analyses described three different T6SS loci present in the pathogenic strain of *P. ananatis* LMG 20103. This initial investigation has been extended to include an additional seven sequenced strains of *P. ananatis* together with 39 strains from different ecological niches. Comparative and phylogenetic analyses were used to investigate the distribution, evolution, intra-strain variability and operon structure of the T6SS in the sequenced strains.

**Results:**

Three different T6SS loci were identified in *P. ananatis* strain LMG 20103 and designated PA T6SS 1-3. PA T6SS-1 was present in all sequenced strains of *P. ananatis* and in all 39 additional strains examined in this study. In addition, PA T6SS-1 included all 13 core T6SS genes required for synthesis of a functional T6SS. The plasmid-borne PA T6SS-2 also included all 13 core T6SS genes but was restricted to only 33% (15/46) of the strains examined. In addition, PA T6SS-2 was restricted to strains of *P. ananatis* isolated from symptomatic plant material. This finding raises the possibility of an association between PA T6SS-2 and either pathogenicity or host specificity. The third cluster PA T6SS-3 was present in all strains analyzed in this study but lacked 11 of the 13 core T6SS genes suggesting it may not encoded a functional T6SS. Inter-strain variability was also associated with *hcp* and *vgrG* islands, which are associated with the T6SS and encode a variable number of proteins usually of unknown function. These proteins may play a role in the fitness of different strains in a variety of ecological niches or as candidate T6SS effectors. Phylogenetic analysis indicated that PA T6SS-1 and PA T6SS-2 are evolutionarily distinct.

**Conclusion:**

Our analysis indicates that the three T6SSs of *P. ananatis* appear to have been independently acquired and may play different roles relating to pathogenicity, host range determination and/or niche adaptation. Future work will be directed toward understanding the roles that these T6SSs play in the biology of *P. ananatis*.

## Background

*Pantoea ananatis* is a Gram-negative, motile, facultative anaerobe belonging to the gamma Proteobacteria. This bacterium can survive and multiply in a variety of ecological niches as a saprophyte, endophyte, epiphyte and pathogen [1]. In its latter role, *P. ananatis* infects a wide range of economically important plants. In South Africa the pathogen causes disease on maize, onion and *Eucalyptus* spp. [2-5] but has also been reported to infect pineapple [6], rice [7], melon [8], sudan grass and sorghum [9,10]. The mechanism of spread of *P. ananatis* between host plants is largely unknown. However, Walcot *et al*. [11] isolated virulent strains of *P. ananatis* from onion seeds, which went on to produce disease symptoms on susceptible onion plants [11]. In addition, Gitaitis *et al*. [12] demonstrated that tabacco thrips (*Frankliniella fusca*) were able to transmit *P. ananatis* into 52% of onion plants analysed [12]. These studies show that seeds and insect vectors are important sources of inoculation and could serve as vehicles for the spread of *P. ananatis* to different geographical regions. Current control measures are limited to cultivation of resistant plant cultivars, eradication of infected plant material and/or the use of biocontrol in the form of lytic phages [1,13,14]. Despite the wide geographical and host range of *P. ananatis*, there is limited information on the genetic determinants of virulence and ecological fitness of the species.

To date, seven different secretion systems have been described in bacteria; namely type I-VII [15,16]. These secretion systems release factors that modulate the host environment to favour bacterial fitness and, in some cases, virulence. The type VI secretion system (T6SS) was first described by Pukatzki *et al*. [17] in *Vibrio cholerae* and was shown to be required for virulence against amoeba and macrophages [17]. This secretion system consists of 15-23 different proteins, which assemble to form an injectisome-like structure similar to an inverted contractile phage particle [18-20]. The T6SS has since been identified in the genome of several pathogenic but also non-pathogenic Gram-negative bacteria, suggesting that it may be involved in functions unrelated to pathogenicity [21-24]. The role of the T6SS in virulence, symbiosis, biofilm formation and stress has been documented in several bacteria [25-30]. In addition, the T6SSs of *Pseudomonas aeruginosa*, *Vibrio cholerae*, *Pseudomonas fluorescens*, *Pseudomonas protegens, Burkholderia thailandensis* and *Serratia marcescens* have been shown to secrete bactericidal effectors which inhibit growth of bacterial species that lack the cognate immunity protein [31-40]. Similarly, the T6SSs of *Pseudomonas syringae* pv. *tomato* DC3000, *Acinetobacter baumannii*, *Acinetobacter baylyi, Vibrio parahaemolyticus* and *Citrobacter rodentium* have also been to play a role in inter-bacterial competition [41-44]. Some bacteria encode more than one evolutionarily distinct T6SS in their genome [23]. Multiple T6SS gene clusters found in a given bacterial genomes are believed to have been acquired by independent horizontal gene transfer events, possibly to play different roles in the biology of different bacteria [23,24,45].

This study focused on the T6SS of the pathogen *Pantoea ananatis*. To date, the genome sequences for eight strains of *P. ananatis* are available, representing both pathogenic and non-pathogenic strains. Type II, type III and type IV secretion systems are well documented virulence determinants of several human and plant pathogens [15,16], although genome mining showed that they were all absent from the genome of *P. ananatis* strain LMG 20103 [1,46]. These findings raise the possibility that the T6SS of *P. ananatis* could play a role in either virulence or ecological fitness of the species. Previous comparative studies identified three distinct T6SS loci on the genome of a single *Eucalyptus* pathogenic *P. ananatis* strain LMG 20103 [46-48]. Thus to better understand the distribution of the T6SS in this versatile pathogen we carried out an in-depth comparative analysis of the T6SS in eight sequenced strains, of which three were recently sequenced as part of this study. To this end, we analyzed the gene content, sequence similarity, synteny, operon structure, and possible evolutionary history of each T6SS loci. PCR and dot blot hybridisations were used to study the distribution of the T6SS in 39 additional strains from a variety of niches. This study represents the first detailed intra-species comparative analysis of the T6SS in *P. ananatis*.

## Results and discussion

### *In silico* identification of T6SS gene clusters in *P. ananatis*

The 13 conserved gene components of the T6SS from *P. ananatis* strain LMG 20103 [48] were used as a bait to identify the T6SS in all sequenced strains of *P. ananatis.* BLASTN and BLASTP searches were done using all eight currently available genomes of *P. ananatis*, which included the LPP-1 megaplasmid found in all sequenced strains [49]. In this manuscript we have used the standard T6SS gene nomenclature proposed by Shalom *et al*. [50]. Based on this nomenclature, the conserved T6SS genes were designated *tss* A-M/ (Type Six Secretion A-M), while the accessory or non-conserved T6SS genes were designated *tag* A-P (Type Six Associated Genes A-P) [50].

**Figure 1:**
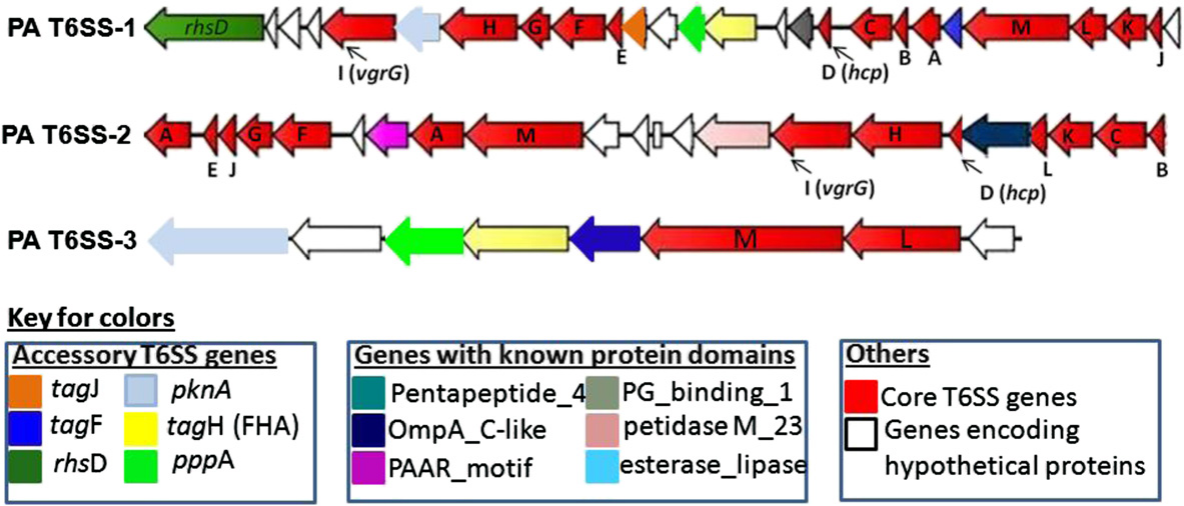
**Genetic organization of the different type VI secretion gene clusters (T6SS) in *Pantoea ananatis *(PA).** Genes are indicated by arrows and the direction of the arrows represents the direction of transcription of the gene related to the rest of the genome. We used the type VI secretion system gene nomenclature of Shalom *et al*. [50]. Conserved core gene components of the T6SS (*tssA-M*) are indicated in red while non-conserved genes associated with the T6SS of limited bacteria (*tagA-P*) are indicated in different colors. The *tag* genes found in the T6SS gene clusters of other bacteria are indicated in the key. The figure is not drawn to scale, PA T6SS-1 and PA T6SS-2 are both over 30 kb and contain up to 20 genes each, while PA T6SS-3 (9 kb) contains 8 genes in all sequenced strains.

Homologs of the T6SS genes were clustered in two distinct genomic regions in all sequenced strains of *P. ananatis*. These regions were designated PA T6SS-1 and PA T6SS-2 (*Pantoea ananatis* T6SS 1, 2). The PA T6SS-1 gene cluster was located on the genome of all eight sequenced strains of *P. ananatis* while PA T6SS-2 gene cluster was located on a ~30 kb region of the LPP-1 megaplasmid of strains AJ13355, LMG 20103 and PA-4. This 30 kb region was missing from the LPP-1 plasmid of *P. ananatis* strains LMG 2665^T^, LMG 5342, BD442, B1-9 and PA-13. We also found that PA T6SS-1 and PA T6SS-2 gene clusters contained all 13 core gene components of the T6SS [23,24]. In addition, we also identified a 9 kb region containing homologs of *icmF* and *dotU* in all eight sequenced strains of *P. ananatis*. This gene cluster, was designated T6SS-3 and was found to be missing the remaining 11 core gene components of the T6SS. Whether or not PA T6SS-3 gene cluster encodes a functional T6SS or represents a truncated T4SS is currently unknown. However, this putative PA T6SS-3 gene cluster was included in this study for purposes of comparative analysis. The overall genetic organisation of each T6SS of *P. ananatis* is presented in Figure 1. The list of all core and accessory T6SS gene components found in the T6SS gene clusters of *P. ananatis*, including their putative functions and COG classification is presented in Table 1.

**Table 1:** **List of core gene and accessory components of *Pantoea ananatis *** **the type VI secretion system (T6SS) and putative function (Pukatzki *et al*.,**[17]**, Zheng and Leung**[28]**, Filloux *et al*.,**[18]**, Bingle *et al*.,**[23]**)**

**Gene**	**Homologues**	**COG classification**	**Putative function**
*tssI*	*vgrG*	COG3501	Effector/structure: forms the T6SS piercing structure
*tssD*	*hcp*	COG3157	Effector/Structure: Homologous to T4 phage tube
*tssC*	*impC, vipB*	COG3517	Homolous to T4 phage contractile tail sheath proteins
*tssB*	*impB,vipA*	COG3516	Homolous to T4 phage contractile tail sheath proteins
*tssH*	*clpV ,vasG*	COG0542	ATPase /effector chaperon/recycling TssB/C
*tssM*	*vasK , icmF*	COG3523	Anchoring T6SS to cell wall
*tssL*	*ompA/dotU*	COG3455	Anchoring T6SS to cell wall
*tssJ*	*vasD ,lip*	COG3521	Anchoring T6SS to cell wall
*tssE*	*impF,vasS*	COG3518	Essential baseplate protein similar toT4 phage gp25 proteins
*tssG*	*impH ,vasB*	COG3520	Unknown function
*tssF*	*impG ,vasA*	COG3519	Unknown function
*tssA*	*impA/vasJ*	COG3515	Unknown function
*tssK*	*impJ ,vasE*	COG3522	Unknown function
*tagB*	*BB0796*	COG1357	Protein with a pentapeptide_4 domain, unknown function
*tagAB*	*BB0795*	COG1357	Protein with a pentapeptide_4 domain, unknown function
*tagE*	*pknA/ppkA*	COG0515	Serine/threonine kinase, post-translational regulation
*tagF*	*impM, sciT*	COG3913	Unknown function
*tagG*	*pppA*	COG0631	Serine/threonine phosphatase, post-translational regulation
*tagH*	*impI*	COG3456	FHA domain-containing protein, post-translational regulation
*tagJ*	*impE*	COG4455	Unknown function
*tagL*	*c3389*	COG2885/COG1360	Protein with an OmpA_C-like domain, unknown function
-	*VCA0105*	-	Protein with a PAAR_motif associated with VgrG piercing structure
-	-	COG3409	Protein with a peptidoglycan binding domain, putative effector
-	*L376_02862*	-	Protein with a peptidase M_23 domain, putative endopeptidase effector
-	*Ebc_4130*	-	Protein with an esterase_lipase domain, unknown function

*Tss* (type VI secretion) genes refers to the T6SS gene nomenclature propose by Shalom *et al*. [50]. These genes have been shown to be essential for secretion of at least two proteins, Hcp and VgrG and are conserved in the genome sequence of over 100 different bacteria encoding a T6SS similar to the prototype described by Pukatzki *et al*. [17].

### Operon structure of the T6SS

The T6SS encoded by most bacteria is organized in discreet transcriptional units or operons [51,52], suggesting coordinated expression [23,24]. We therefore, investigated the organization of conserved genes in the *P. ananatis* T6SS. The core genes of PA T6SS-1 were clustered in three highly conserved operons; group 1 (*tssJ-tssK-dotU-icmF*) group 2 (*tssB-tssC*-*hcp*) and group 3 (*tssE-tssF-tssG-tssH*). PA T6SS-2 showed a considerable level of gene shuffling compared to PA T6SS-1, with gene order being highly variable between each of the different groupings. The consensus grouping in PA T6SS-2 included *tssB-tssC-tssK-dotU* and *tssF-tssG-tssJ-tssE,* while *tssH-vgrG* and *icmF-tssA* were stand-alone operons linked to non-conserved T6SS genes. The start and stop codons of all 9 genes located in PA T6SS-3 gene cluster overlapped with each other, suggesting that PA T6SS-3 represents a single transcriptional unit. These different operon structures suggested the independent acquisition of the T6SS clusters, each of which may play a different role in the biology of *P. ananatis*.

### Distribution of the PA T6SS in other strains of *P. ananatis*

#### PCR and dot blot analysis

To determine the prevalence of the three PA T6SS clusters [1-3] among *P. ananatis* strains, we analyzed the distribution of each T6SS cluster in 46 different strains of the pathogen. BlastP analysis showed that the gene products of PA T6SS-1 and 2 shared less than 50% amino acid similarity (Additional file 1: Table S1). This low sequence similarity allowed specific PCR primers to be designed within conserved regions located on the DNA sequence of the targeted genes. For primer design, the targeted T6SS gene homologs from eight sequenced strains of *P. ananatis* were aligned in BioEdit using ClustalW multiple alignment program and PCR primers designed within the conserved DNA regions located on these genes. BlastN analysis showed that the conserved regions used for primer design were not present on the DNA sequences of T6SS gene homologs found in other clusters. We, therefore, saw no cross reactivity during the PCR amplification. The list of primers and strains of *P. ananatis* used for PCR analysis is provided in Additional file 2: Table S2 and Additional file 3: Table S3, respectively.

PCR amplification showed that the T6SS-1 and T6SS-3 homologs were present in all tested strains of *P. ananatis*. PCR amplification using PA T6SS-2 gene specific primers identified homologs in only 15 of the 46 strains of *P. ananatis* (33%) tested (Additional file 4: Table S4). The distribution of PA T6SS-2 was further confirmed by dot blot hybridization using probes for *tssA*, *tssE*, *tssJ* and *tssK* (results not shown). Our results showed that PA T6SS-2 was present in strains of *P. ananatis* isolated from symptomatic maize, onion, pineapple fruit and *Eucalyptus* spp. However, not all strains of *P. ananatis* isolated from the same host plant contained PA T6SS-2. The cluster was only found in 3 of the 6 strains of *P. ananatis* (50%) isolated from either maize, onion, pineapple and *Eucalyptus* spp., while the remaining 3 strains, isolated from the same host but different plants, did not have the cluster. In addition, PA T6SS-2 was absent in all strains of *P. ananatis* isolated from symptomatic wheat (2 isolates), honeydew melon (4 isolates), rice (7 isolates) and sugarcane (1 isolate). These results suggest that PA T6SS-2 could be a host range or virulence determinant of *P. ananatis*. Future studies will undertake pathogenicity and cross inoculation trials to validate this correlation. In addition, the cluster was also found in *P. ananatis* strains AJ13355 and Yomogi-101 which have been shown not to cause disease on a range of host plants ([53,54], and data not shown), suggesting that PA T6SS-2 could be involved in other processed related to the ecological fitness of the species.

### Comparative analysis of T6SS gene clusters from different strains of *P. ananatis*

**Figure 2:**
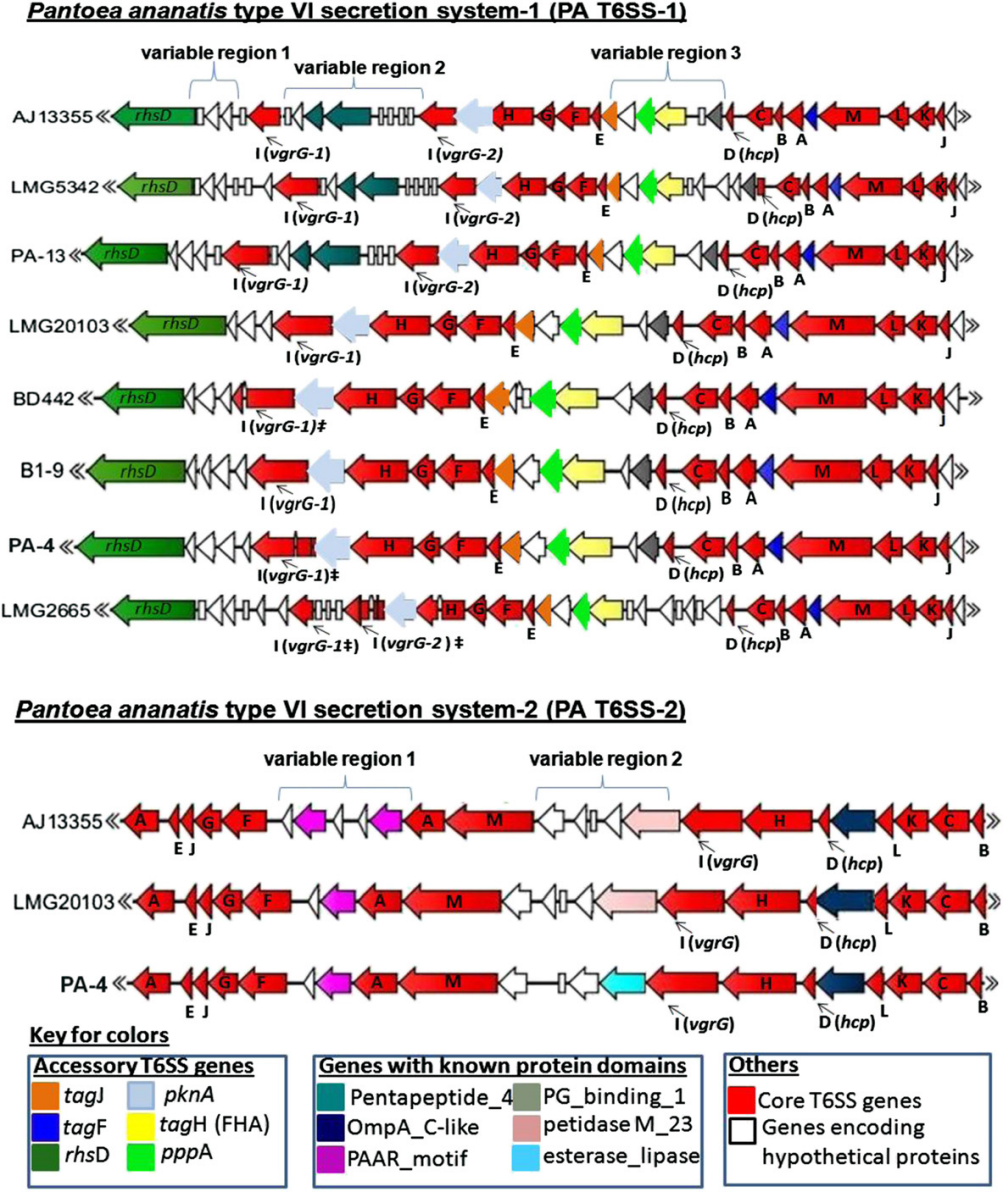
**Comparison of the *Pantoea ananatis *type VI secretion systems 1 and 2 (T6SS-1 and T6SS-2).** All 13 conserved core gene components of the T6SS are indicated in red while non-conserved genes associated with the T6SS of limited bacteria are indicated in different colors. The letters in the figure represent conserved T6SS genes based on the nomenclature of Shalom *et al*. [50]. PA T6SS-1 was found in all eight sequenced strains analyzed, while PA T6SS-2 was restricted to AJ13355 (saprophyte), LMG 20103 (pathogen of *Eucalyptus* spp) and PA-4 (onion pathogen). ‡ represents genes found in two unassembled contigs in PA T6SS-1.

Homologous PA T6SSs encoded by different strains of *P. ananatis* were highly conserved in terms of sequence similarity, gene content and operon structure (Figure 2 and Additional file 5: Figure S1). A detailed description of the genes found in individual T6SSs encoded by all sequenced strains of *P. ananatis* analyzed in this study are provided in (Additional file 6: Table S5-S12 and Additional file 7: Table S13-S15).

1) PA T6SS-1

**Figure 3:**
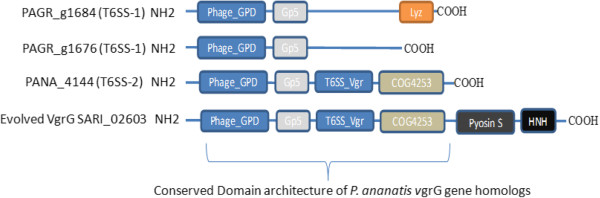
**Homolog of *vgrG *genes found in the type VI secretion system gene clusters of sequenced strains of *Pantoea ananatis*.** Domains are represented in different colors. *P. ananatis* strain PA-13 has two structurally different *vgrG* genes (PAGR_g1684 and PAGR_g1676) within T6SS-1. Lyz = lysozyme / Beta-N-acetylglucosaminidase domain is found in PAGR_g1684 which is missing from the *vgrG* homolog (PAGR_g1676). PANA_4144 found in T6SS-2 of strain LMG 20103 has a domain architecture similar to part of SARI_02603 of *Salmonella enterica* subspecies *arizonae*.

The genetic architecture of PA T6SS-1 was shown to be conserved amongst all sequenced strains of *P. ananatis*. The *tssD* (*hcp*) and *tssI* (*vgrG*) genes found in this cluster encoded Hcp and VgrG proteins that do not have C-terminal extensions as found in “evolved” VgrG and Hcp proteins [45,55,56]. The C-terminal extension of some evolved VgrG proteins, such as VgrG1 of *V. cholerae* and *Aeromonas hydrophila*, have been associated with actin cross-linking and actin ADP ribosylation activity in mammalian host cells, respectively [55,56]. *P. ananatis* strains PA-4, BD442, B1-9 and LMG 20103 had a single *vgrG* gene, while strains AJ13355, PA-13, LMG 5342 and LMG 2665^T^ had an additional copy of *vgrG*. These *vgrG* genes appear to encode VgrG proteins with different domain architectures, characterized by the presence or absence of a C-terminal Beta-N-acetylglucosaminidase domain (Figure 3). This C-terminal domain is associated with lysozymes belonging to the glycoside hydrolase family 73 (PF01832) [57,58]. It is possible, therefore, that the different VgrG proteins encoded by each *vgrG* gene are mobilized to the T6SS baseplate under different physiological conditions or play different roles either as effectors, structural elements or both.

**Figure 4:**
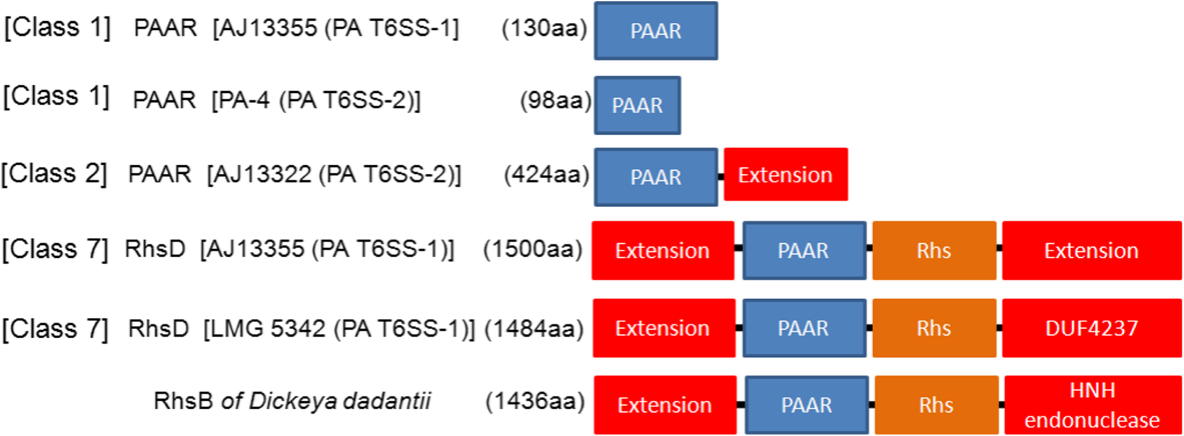
**Domain architecture of PAAR repeat proteins associated of *Pantoea ananatis *type VI secretion system 1 and 2 (T6SS-1 and T6SS-2).** We used the nomenclature of Shneider *et al*. [59], which categorized PAAR proteins into different classes [1-7] based on domain architecture. The different domains and extensions associates with *P. ananatis* PAAR proteins are indicated by different colors. No putative effector domains were associated with N or C-terminal extensions located in most PAAR repeat proteins of *P. ananatis*. However, the RhsD protein of LMG 5342, LMG 2665^T^ and B1-9 showed extensive structural homology to the insecticidal YenC2 toxins of *Yersinia entomophaga.* The domain architecture of *P. ananatis* RhsD protein is similar to the RhsB endonuclease toxin of *Dickeya dadantii.*

Regions associated with *hcp* and *vgrG* contain genes that encode a variable number of accessory and hypothetical proteins that account for strain specific differences. The first variable region in PA T6SS-1 is located between the major *rhsD* element and *vgrG*. Genes found in this region encode mostly hypothetical proteins and proteins with either a PAAR (proline-alanine-alanine-arginine) repeat or pentapeptide_4 domains. PAAR repeat proteins of bacteria have categorised into different classes (Class 1-7) based on their domain architectures [58]. PAAR repeat proteins of several bacteria have effector domains on the N or C-terminal [58]. Some of these effector domains include: transthyretin, lipase, nuclease, deaminase, and ADP-ribosyl transferase [58]. The genes products of PA-13 PAGR_g1683 and LMG 5342 PANA5342_1748 belong to the Class 1 PAAR domain architecture proteins and share 100% sequence similarity (Figure 4). In addition, PAGR_g1683 and PANA5342_1748 have no N or C terminal extensions and showed structural homolog to the PAAR repeat protein of *V. cholerae* (4jiv_D–Hhpred score 105.4). A recent study showed that the PAAR repeat proteins of *E. coli* and *V. cholerae* bind to the Gp5-VgrG complex by means of non-covalent interactions [59]. In addition, PAAR repeat proteins of *V. cholerae* and *Acinetobacter baylyi* were shown to be bactericidal effectors associated with T6SS-mediated killing of *E. coli*[59]. These findings have led to the speculation that, PAAR repeat proteins carrying different effector domain located on either their N or C-terminal extensions may also bind to the VgrG spike and mediate secretion of these effectors by the T6SS [59,60]. It is also speculated that PAAR repeat proteins may form non-covalent interactions with different effectors, thereby recruiting them to the T6SS spike complex [59]. It is, therefore, possible that the PAAR repeat proteins encoded by genes located in the *vgrG* island of *P. ananatis* T6SS-1 gene cluster may either be T6SS effectors associated with inter-bacterial competition or may mediate secretion of other effectors.

**Figure 5:**
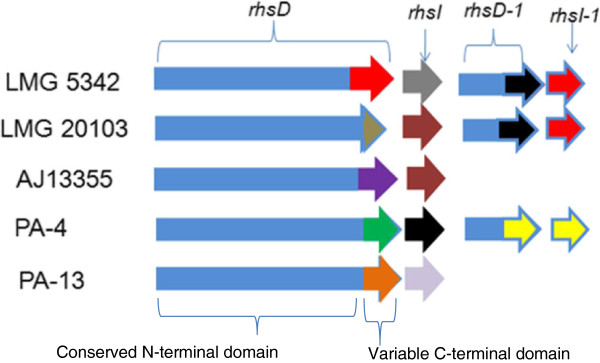
**Genetic organization of the different *rhs *loci associated with the type VI secretion system 1 gene cluster (T6SS-1) of *Pantoea ananatis*.** Representative strains of *P. ananatis* with distinct *rhs* loci are indicated in the figure. Strains LMG 5342, B1-9 and LMG 2665^T^ have identical *rhsD*/*rhsI* gene homologs. Similarly, strain PA-4 and BD442 also have identical *rhsD*/*rhI* gene homologs. Conserved and variable regions associated with *rhsD* genes are indicated in the figure. The *rhsI* gene is located downstream of the *rhsD* gene and the colors indicate the different *rhI* genes encoded by different strains of *P. ananatis*. The figure is not drawn to scale.

PAAR-repeat domains are also found in the *rhsD* gene of PA T6SS-1. A recent study, aimed at identifying polymorphic toxins in bacterial genomes using comparative analysis, sequence and structural analysis, identified RhsD as putative T6SS effectors of *P. ananatis*, based on the presence of PAAR repeats on this protein [59]. Similarly, the RhsD protein of *Serratia marcescens* was recently identified in a T6SS secretome analysis, suggesting that this protein is secreted by the T6SS [40]. Bacteria Rhs proteins have been associated with different phenotypes such as social motility, inflammasome-mediated cell death, virulence in mice, insecticidal toxin production, polysaccharide transport and bacteriocin production [61-69]. In addition, the *rhsA* and *rhsB* genes of *Dickeya dadantii* encode endonuclease toxins which have been associated with contact-dependent killing of other bacteria species [70,71]. Toxin producing strains of *D. dadantii* also express the cognate immunity factors from the *rhsI* gene located downstream of the *rhsA* and *rhsB* genes [70,71]. The *rhsD* locus of *P. ananatis* has a genetic organization similar to that of *D. dadantii* consisting of the *rhsD* gene which is followed by the *rhsI* homolog (Figure 5). In addition, *P. ananatis rhsD* genes have a conserved N-terminal domain and a variable C-terminal domain which is characteristic of several bacteria Rhs toxins (Additional file 8: Figure S2) [72,73]. We identified a DUF4237 domain of unknown function on the C-terminal extensions of RhsD proteins of *P. ananatis* strains B1-9, LMG 2665^T^ and LMG 5342, which is missing from the remaining strains. Furthermore, the RhsD proteins of strains B1-9, LMG 2665^T^ and LMG 5342 showed extensive structural homology to the insecticidal YenC2 toxin of *Yersinia entomophaga* (4igl_B–Hhpred score 574, 559.5 and 573.4, respectively) [65,74]. These findings suggest that the *rhsD* loci associated with the T6SS-1 gene clusters of *P. ananatis* may encode different toxin/immunity factors which play different roles as either bactericidal or insecticidal toxins. Alternatively, RhsD proteins of *P. ananatis* may mediate secretion of other T6SS effectors which are non-covalently associated with either their N or C-terminal extension, as predicted for other PAAR proteins [59].

Homologs of *tagAB* and *tagB,* which encode pentapeptide repeat proteins were also identified in the *vgrG* islands of *P. ananatis* strains PA-13, AJ13355 and LMG 5342. In strain PA 13, the pentapeptide repeat proteins are encoded by PAGR_g1680 and PAGR_g1681 which correspond to *tagAB* and *tagB* genes, respectively. Sequence analysis performed on PAGR_g1680 and PAGR_g1681 predicted no signal sequence and no transmembrane helices [75,76]. PsortB analysis predicted the gene product of PAGR_g1680 to be a secreted effector while the sub-cellular localization of PAGR_g1681 protein is unknown [77]. Homologs of *tagAB* and *tagB* genes have also been in identified in the T6SS gene clusters of *Serratia marcescens, Azotobacter vinelandii, Burkolderia bronchiseptica,* including the T6SS-5 gene clusters of *B. mallei, B. pseudomallei* and *B. thailandensis*, however, these genes have not been functionally characterised and the biological function of their gene products is currently unknown [30,38,50].

The second variable region found in *P. ananatis* T6SS-1 gene cluster is located between *hcp* and *tagH* and contains a gene encoding a protein with a peptidoglycan binding domain (PG_binding). In strain LMG 5342, the protein is encoded by PANA5342_1731 and showed structural homology to the lytic transglycosylase enzyme of *Pseudomonas aeruginosa* bacteriophage phiKZ (3bkh_A Hhpred–score 146) while the corresponding homologs found in strains LMG 20103, PA-4, B1-9 and AJ13355 showed structural homology to the pesticin toxin of *Yersinia pestis* (4aqm_A Hhpred–score 290.2, 283.4, 290.2 and 283.4, respectively). Pesticin and lytic transglycosylase enzymes are bactericidal effectors which degrade peptidoglycan by cleaving the β-1,4 glycosidic bond between *N*-acetylmuramic acid and the *N*-acetylglucosamine moieties [78-82]. These findings suggest that genes found in the *hcp* island of *P. ananatis* T6SS-1 cluster may encode bactericidal effectors which are functionally related to the Type VI secretion glycoside hydrolase effectors 1-3 (Tge1-3) [37,83]. Genes encoding proteins with the PG_binding domain were not identified in the T6SS-1 *hcp* island of *P. ananatis* strain LMG 2665^T^. However, the gene product of LMG 2665^T^ N454_00628 showed weak structural homology to Colicin S4 of *Escherichia coli* (3few_X–Hhpred score 55.8). Colicin S4 is a pore-forming bacteriocin which kills bacteria species not expressing the immunity factor [84,85]. It is, therefore, possible that the gene product of LMG 2665^T^ N454_00628 may be a bacteriocidal effector which plays a role in inter-bacterial competition. We did not identify homologs of Type VI amidase 1-4 (Tae 1-4), Type VI lipase effectors 1-5 (Tle 1-5), Small secreted proteins 1-6 (Ssp 1-6) and Type VI secretion exported 1, 2 (Tse 1, 2) in the T6SS-1 gene cluster of *P. ananatis*, based on sequence and structural analysis [34,36,39,40].

2) PA T6SS-2

The genetic architecture of PA T6SS-2 is highly conserved in all strains that harbor the cluster. PA T6SS-2 was found to contain a single *vgrG* gene that encodes a VgrG protein with an additional C-terminal DUF2345 (COG4253) domain of unknown function. Conserved Domain architecture analysis showed that this domain was conserved in the *vgrG* genes of several different bacteria. All strains of *P. ananatis* that harbor this cluster encode two copies of the *tssA* gene within the cluster showing 22% amino acid similarity. Duplication of the *tssA* gene has also been reported in the T6SS gene clusters of *Vibrio cholerae*, *Escherichia coli* O157:H7, *Yersinia pseudotuberculosis* and *Salmonella enterica* serotype Gallinarum [21,45,50]. The reason for this duplication is unknown, as is the exact role that TssA plays as an essential T6SS structural protein.

Two variable regions were identified in PA T6SS-2, which contained genes encoding non-conserved T6SS components and hypothetical proteins. The first variable region is located between *tssA* and *tssF* and encodes several strain specific hypothetical proteins including proteins with a PAAR_motif. The genes products of LMG 20103 PANA_4136, AJ13355 PAJ_p0154 and PA-4 N455_00706 contain 424 amino acids, sharing 99% amino acid similarity and belong to the Class 2 PAAR domain architecture proteins. Furthermore, these PAAR proteins all contained identical C-terminal extensions with no putative effector domains. The gene products of PANA_4136, PAJ_p0154 and PA-4 N455_00706 may either be T6SS effectors or mediate secretion of other effectors bound to their C-terminal extensions [59,60]. The second variable region in PA T6SS-2 gene cluster is located between *icmF* and *vgrG*. This region in LMG20103 contains four genes (PANA_4140 to 4143) encoding hypothetical proteins with homologs present in strain AJ13355 but is missing from strain PA-4. PANA_4143 encodes a protein with a peptidase_M23 domain. This protein is a putative endopeptidase and is predicted to be a T6SS effector based on its high structural homology to the secreted chitinase G of *Streptomyces coelicolor*[48,86]. The corresponding variable region in *P. ananatis* strain PA-4 contains a gene with an esterase_lipase domain and belongs to the family lecithin: cholesterol acyltransferase (PF02450). This protein is predicted to be involved in extracellular metabolism of plasma lipoproteins, including cholesterol [87]. Genes with an esterase_lipase domain have been found in the vicinity of *vgrG* genes outside the major T6SS of *Pseudomonas* spp. In *P. aeroginosa* these genes form part of the “cargo” genes predicted to have been acquired by recent horizontal gene transfer [88]. In addition, the N455_00710 gene which is located in the *vgrG* island of strain PA-4 T6SS-2 cluster, encodes a Class 1 PAAR domain architecture protein containing 98 amino acids with no N or C-terminal extensions. This PAAR protein may play a role as a T6SS effector associated with inter-bacterial competition [59,60]. Homologs of functionally characterized T6SS effectors such as type VI lipase effectors 1-5, type VI amidase 1-4, type VI secretion glycoside hydrolase effectors, Small secreted proteins 1-6 and Type VI secretion exported 1-2, were not identified in *P. ananatis* T6SS-2 gene cluster, using sequence analysis and structural homology search tools.

3) PA T6SS-3

Comparative analysis of PA T6SS-3 showed that there was no variability of this cluster between the different strains of *P. ananatis*. The genetic architecture, gene order and gene content of PA T6SS-3 was conserved in all sequenced strains of *P. ananatis*. Interestingly, all genes found in PA T6SS-3 showed high sequence similarity to genes found in *Pantoea* sp. At-9b, *Pantoea* sp. aB-valens, *P. vagans* C9-1, *P. agglomerans* E325 and *Erwinia billingiae* Eb661. These *Pantoea* and *Erwinia* spp. have a homologous cluster highly similar to PA T6SS-3 in terms of gene content and operon structure [48]. The high conservation of this cluster suggests a strong selective pressure to maintain the gene content and order, although its specific role is unknown. The fact that the cluster is missing 11 core gene components of the T6SS suggests that this system does not encode a functional T6SS, although this is yet to be confirmed.

### “Orphan” Hcp and VgrG proteins

When the T6SS genes *hcp* and *vgrG* are present outside the T6SS they are often referred to as “orphan” *vgrG* and *hcp* genes. Our analysis did not identify “orphan” *vgrG* genes in any of the sequenced genomes of *P. ananatis*. However, we identified three different “orphan” *hcp* genes in *P. ananatis* strain PA-13 (PAGR_g1583, PAGR_g1584 and PAGR_g3636). These “orphan” *hcp* genes were designated *hcp*-1, *hcp*-2 and *hcp*-3 to correspond to PAGR_g1583, PAGR_g1584 and PAGR_g3636, respectively. Genes *hcp-*1 and *hcp*-2 are adjacent to each other and have homologs present in all sequenced strains of *P. ananatis,* while *hcp*-3 is restricted to *P. ananatis* PA-13. Multiple alignments of the amino acid sequences of representative *P. ananatis* Hcp proteins showed that Hcp-3 protein is highly divergent from Hcp-1, Hcp-2 and T6SS-associated Hcp proteins (Additional file 9: Figure S3). All “orphan” *hcp* genes in *P. ananatis* are found in the vicinity of genes encoding hypothetical proteins and a putative endoribonuclease SymE, which is part of an SOS inducible toxin/antitoxin system [89]. It remains to be determined whether “orphan” *hcp* genes of *P. ananatis* are association with the major T6SSs, as either T6SS effectors or structural proteins, or whether the conserved association between *symE* and “orphan” *hcp* genes is important in other aspects of the biology of *P. ananatis*.

### Phylogenetic analysis of the T6SS

**Figure 6:**
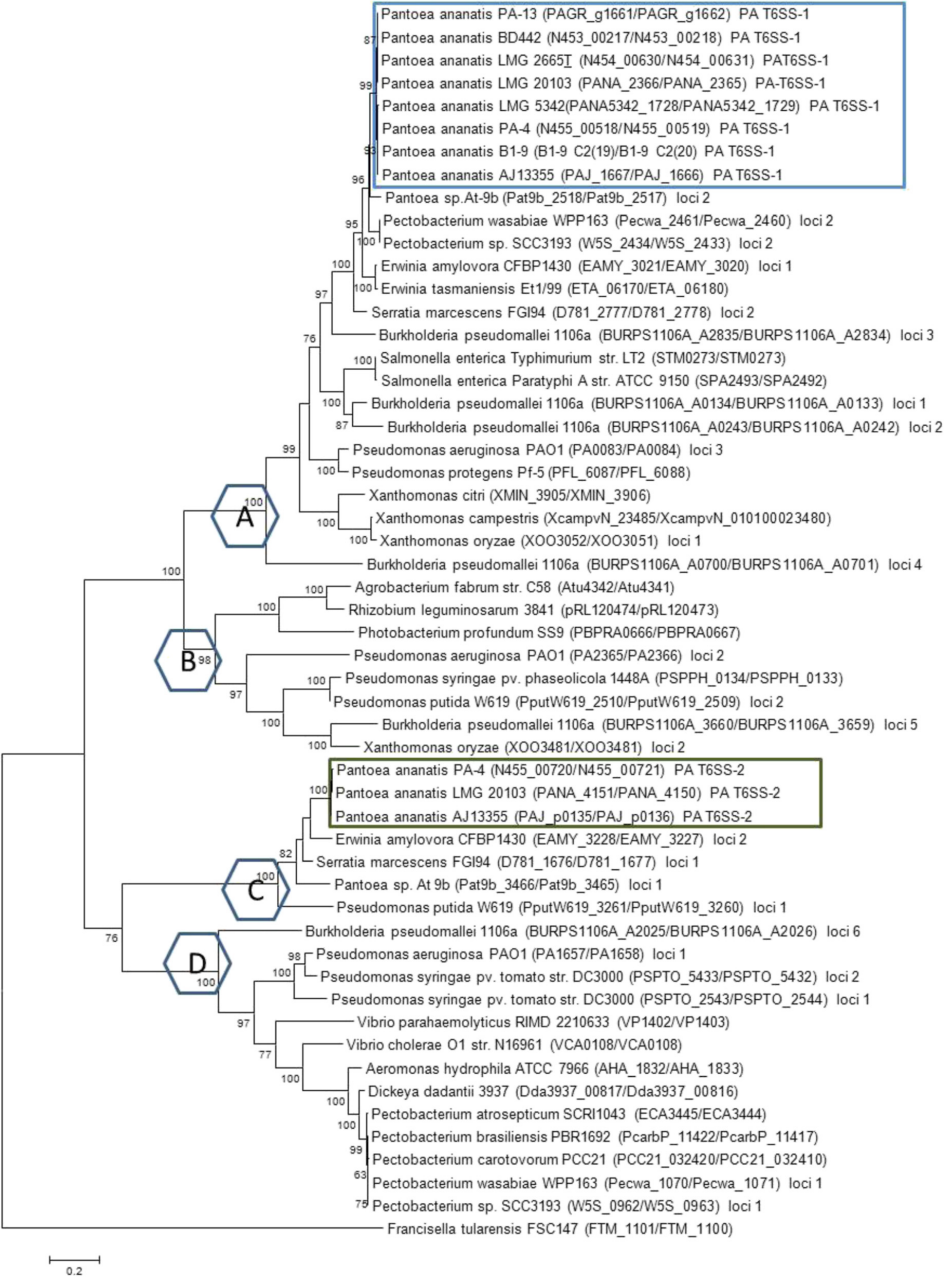
**Evolutionary relationships of the different type VI secretion systems of *Pantoea ananatis *using concatenated TssB and TssC amino acid sequences.** Phylogenetic analysis were conducted in MEGA6 [90]. The amino acid substitution model was determined by ProtTest [91]. The evolutionary history was inferred using the Maximum Likelihood method based on the Le and Gascuel (LG) + G + F model [92]. The percentage of replicate trees in which the associated taxa clustered together in the bootstrap test (1000 replicates) is shown next to the branches. The tree is drawn to scale, with branch lengths measured in the number of substitutions per site. The TssB/C homologs used in this study are indicated next to the name of the bacteria. Blue octagons represent T6SS phylogenetic Groups A-D proposed by Bingle *et al*. [23]. Representative position of PA T6SS-1 and PA T6SS-2 are shown in blue and green rectangles, respectively. Details of all bacteria used in are given in (Additional file 10: Table S16).

Phylogenetic analysis was used to infer the evolutionary history of the T6SS using the Maximum Likelihood Method base on the Le and Gascuel (LG) + G + F amino acid substitution model, as determined by ProtTest [90-92]. Representative bacteria from the different T6SS phylogenetic groups A-D were included in the analysis [23,45] (Additional file 10: Table S16). The analysis showed that PA T6SS-1 belonged to phylogenetic Group A, while PA T6SS-2 belonged to Group C (Figure 6). PA T6SS-1 was phylogenetically closest to T6SS loci 1 of *Pantoea* sp At-9b (an insect endophyte), while PA T6SS-2 was closest to T6SS loci 2 of *Erwinia amylovora* CFBP1430 (a plant pathogen). Our phylogenetic analysis resembled the analysis performed by Bingle *et al*. [23] which showed that phylogenetic Group A to D contained pathogenic and non-pathogenic bacteria associated with different ecological niches [23]. Similarly, functionally characterized T6SS with a known role in virulence or inter-bacterial competition were represented in the different Groups. For example, the H1-T6SS of *P. aeruginosa* which is known secreted Tse1 to 3 antimicrobial effectors was found in Group A while the T6SSs of *Vibrio cholerae* and *Peudomonas. syringae* pv *tomato* with a similar role in inter-bacterial competition belonged to Group D [31,32,41]. In addition, all four phylogenetic Groups contained bacteria T6SSs which have been associated with virulence. Together, these finding suggest that bacteria T6SSs found in phylogenentic Group A to D are evolutionarily distinct and play different roles in pathogenic and non-pathogenic bacteria [23,24].

Our phylogenetic tree also showed that *P. ananatis* T6SS-1 and T6SS-2 clustered away from the T6SS of several important plant pathogens such as *Xanthomonas citri*, *X. campestris*, *X. oryzae, Pseudomonas syringae* pv. *syringae, Pseudomonas syringae* pv. *phaseolicola*, *Pectobacterium atrosepticum* SCRI1043, *Pectobacterium carotovorum* subsp. *carotovorum* and *Pectobacterium carotovorum* subsp. *brasiliensis*. Functional studies have shown that the T6SS gene clusters of *P. atrosepticum* and *P. syringae* are only partially required for disease development. The T6SS mutants of these bacteria were either only slightly reduced in the ability to cause disease or caused disease symptoms on susceptible host plants similar to the wild type strains [41,93-96]. These findings, together with our T6SS phylogenetic groupings, suggests that 1) the T6SS of different plant pathogens were acquired from unrelated bacteria or distantly-related ancestors, 2) the T6SS clusters found in different phytopathogens may play different roles depending on the host plant or ecological niche, and 3) T6SS-1 and T6SS-2 may play an important role in the virulence of *P. ananatis* in susceptible host plants.

To infer the evolutional history of “orphan” *hcp* genes (*hcp*-1, 2 and 3) in *P. ananatis* we constructed a second Maximum Likelihood tree using the General Reverse Transcriptase (rtRev) + I + G + F amino acid substitution models, as determined by ProtTest [97]. Our phylogenetic analysis also included representative T6SS-associated *hcp* genes and “orphan” *hcp* genes from other bacteria (Additional file 11: Table S17). The resulting phylogeny revealed four distinct clusters, which we have designated as *hcp* Cluster 1-4 to distinguish them from T6SS phylogenetic Group A-D [23] and Group I-V [24]. Our results indicate that *hcp*-1 and *hcp*-2 belong to Cluster 3, together with the T6SS-associated *hcp* genes found in PA-T6SS-1 (Additional file 12: Figure S4). Hcp-3 (PAGR_g3636) belongs to Cluster 2, together with T6SS-associated *hcp* genes present in PA-T6SS-2. Hcp-3, however, forms part of a subset of Cluster 2, and was phylogenetically close to orphan Hcp proteins from *klebsiella varriicola* At-22 and *Erwinia amylovora* ATCC BAA-2158. These *hcp* groupings suggest either independent acquisition of “orphan” *hcp* genes from different ancestors or gene duplication and rearrangement of T6SS-associated *hcp* genes.

## Conclusion

Comparative analyses of the T6SS in the genomes of sequenced strains of *P. ananatis* identified three putative gene clusters PA T6SS-1, PA T6SS-2 and PA T6SS-3. The former two of these were potentially functional as they contained the 13 core genes necessary for synthesis of a functional T6SS [23,24,30]. PA T6SS-1 was widespread in the genome of all sequenced strains including environmental isolates, while PA T6SS-2 was plasmid borne and restricted mostly to pathogenic strains of *P. ananatis* isolated from certain classes of plants. This finding suggests a potential association of PA T6SS-2 with host range determination. However, the finding that PA T6SS-1 and PA T6SS-2 were present in both pathogenic and non-pathogenic strains of *P. ananatis* supports the concept that the T6SS may evolve to play different roles unrelated to pathogenicity, e.g. competition against other microbes, fitness and/or niche adaptation [18,23,27,39]. The genetic organization and phylogenetic groupings of PA T6SS-1 and −2 further suggests that these clusters were independently acquired to play differing roles in the different strains of *P. ananatis*. Furthermore, the variable regions associated with *hcp* and *vgrG* genes could account for specialization of each T6SS based on the needs of the specific strain. In the future, key questions that need to be addressed include determining: 1) whether the T6SSs of *P. ananatis* are functionally active and what roles they play in host-pathogen interactions and fitness; 2) which *in vitro* and *in vivo* conditions activate the T6SSs; 3) the presence of different potential effectors secreted by the T6SSs of *P. ananatis* and their physiological relevance to fitness and host-pathogen interactions; and 4) how T6SSs are regulated in these strains.

## Methods

### *P. ananatis* T6SS data acquisition

The annotated genome sequences of different *P. ananatis* strains were downloaded from NCBI with the accession numbers LMG 5342 (chromosome HE617160.1, plasmid pPANA10 HE617161.1); AJ13355 (chromosome AP012032.1, plasmid AP012033.1); PA-13 (chromosome CP003085.1, plasmid PAGR_pCP003086.1); LMG 20103 (chromosome CP001875.2) and the draft genome of B1-9 (CAEJ00000000.1). The draft genomes of *P. ananatis* BD 442, PA-4 and LMG 2665^T^ were sequenced and partially assembled in our laboratory. The sequences and fully annotated contigs representing the different type VI secretion system gene clusters have been deposited in GenBank for strain BD 442 (KF552073, KF552074), PA-4 (KF590029, KF590030, KF590028) and LMG 2665^T^ (KF590026, KF590027). All eight sequenced strains of *P. ananatis* were obtained from different geographical regions and were isolated from different diseased plants or other environmental samples. Detailed information on the strains is presented in Additional file 3: Table S3.

### *In silico* identification of T6SS cluster

Genes associated with the T6SS, including flanking regions identified in *P. ananatis* strain LMG 20103 by De Maayer *et al*. [46], were used as bait to search for T6SS homologs in all sequenced *P. ananatis* genomes and plasmids using BLASTN and BLASTP [98,99]. Nucleotide sequences representing entire T6SS regions, including flanking regions, were extracted from each genome and used for *de novo* gene prediction using F-GenesB (http://www.Softberry.com). All predicted genes were searched against non-redundant protein databases at NCBI to identify homologs. SignalP 4.0 and TMHMM Server v.2.0 were used to predict signal peptides and trans-membrane helices [75,76]. Protein localization and functional classifications were done using PSORTb [77], InterProScan [100], Conserved domains and CDD domain architecture-Search tool on NCBI [101]. Protein structural homology analyses were done using the HHpred [102]. Proteins were clustered based on their COG groups [103].

### PCR and dot blot hybridisations

A minimum of four primer pairs were designed per T6SS cluster to amplifying locus specific genes. For primer design, the nucleotide sequences for each gene of interest were extracted from all eight sequenced strains of *P. ananatis*. BLASTP analysis showed that T6SS gene products from homologous T6SS cluster found in all sequenced strains of *P. ananatis* were over 98% similar [Additional file 6: Table S9-S12 and Additional file 7: Table S15]. In addition, BLASTP analysis showed that there was less than 70% similarity between gene products found in the different T6SS clusters (Additional file 1: Table S1). The low sequence similarity between gene homologs from in each T6SS cluster, therefore, allowed specific primers to be designed within conserved T6SS gene regions. Importantly, PCR analysis showed that the primers were specific and did not amplify T6SS homologs found in other T6SS gene clusters found in the genome sequence of *P. ananatis*. Genomic DNA was isolated from 46 different *P. ananatis* strains using the Quick-gDNA™MiniPrepkit (ZYMO RESEARCH, USA) following the manufacturer’s instructions. PCR amplification using SuperTherm DNA polymerase (Southern Cross Biotechnology, RSA) was performed with a Veriti^R^ Thermal Cycler (Applied Biosystems, USA). DNA sequencing was done using the ABI PRISM3100 Genetic Analyzer (Applied Biosystems) at the DNA Sequencing Facility (University of Pretoria-RSA). Colony hybridisation was used to validate the PCR results. Probes for hybridisation were labeled with Digoxigenin-11-dUTP using DIG PCR labeling Kit (Roche Applied Science, USA) according to the manufacturer’s instructions. Membrane hybridization, washing and detection were done using DIG DNA labeling and detection kit (Roche Applied Science, USA) as directed by the manufacturer.

### Phylogenetic analyses

Two phylogenetic analyses were carried out, one using concatenated amino acid sequences of TssB and TssC and the second using amino acid sequences of Hcp and “orphan” Hcp. The TssBC analysis showed the phylogenetic grouping of the different T6SSs of *P. ananatis*, while a further phylogenetic grouping examined the relationship between T6SS-associated Hcp and orphan Hcp proteins. Amino acid sequences of TssB, TssC, Hcp and “orphan” Hcp proteins representing bacteria from T6SS phylogenetic Groups A-D were downloaded from NCBI [23,24]. Amino acid sequences were aligned by ClustalW and phylogenetic analysis conducted in MEGA6 [90]. The amino acid substitution models were determined by ProtTest and applied to this study [91]. The evolutionary history of TssB/C and Hcp proteins were inferred by using the Maximum Likelihood method based on 1) the Le and Gascuel (LG) + G + F amino acid substitution model [92] for TssB/C proteins and 2) the General Reverse Transcriptase (rtRev) + I + G + F amino acid substitution model [97] for Hcp proteins.

### Availability of supporting data

The data sets supporting the results of this article are included within the article and its additional files. Alignments and Phylogenetic trees which support the findings presented in this research article are available online in the Dryad Digital Repository (doi: 10.5061/dryad.vd7k7).

## Competing interests

The authors declare that they have no competing interests.

## Authors’ contributions

DYS, LNM, SNV, IKT and TAC conceived the study. DYS performed experiments and analysis. DYS, LNM, SNV, IKT and TAC wrote the original manuscript. All authors read and approved the final version.

## Supplementary Material

Additional file 1: Table S1 BLASTP analysis of type VI secretion system (T6SS) homologs found in *Pantoea ananatis* strain LMG 20103. This table shows the percentage amino acid identify between the T6SS gene products of *P. ananatis* T6SS-1 to 3. The gene products of LMG 20103 T6SS-1 were used as bait for BlastP analysis. Click here for file

Additional file 2: Table S2 List of primers used for PCR amplification of type VI secretion system genes from different strains of *Pantoea ananatis*. This file contains PCR primer sequences and the T6SS genes of *P. ananatis* which were targeted for Polymerase Chain Reaction (PCR) amplification. Click here for file

Additional file 3: Table S3 Strains of *Pantoea ananatis* tested for the presence of T6SS gene clusters. This file contains the list of all strains of *Pantoea ananatis* screened for the presence or absence type VI secretion system gene homologs. The file also contains information on the host of isolation and country from which the different strains of *P. ananatis* were isolated from. Click here for file

Additional file 4: Table S4 Distribution of type VI secretion system gene clusters in 46 strains of *Pantoea ananatis*. This file contains results of the PCR screen, which clearly shows the distribution of type VI secretion system genes in all strains of *Pantoea ananatis* analysed in this study. Click here for file

Additional file 5: Figure S1 Synteny between *Pantoea ananatis* type VI secretion system 1, 2 and 3 (PA T6SS-1, 2, 3). This file contains pairwise alignment of the homologous type VI secretion system gene clusters found in sequenced strains of *P. ananatis*. Alignments were generated using Mauve v.2.3.1 and show variable and conserved regions between the homologous T6SS gene clusters from different strains of *P. ananatis*. Click here for file

Additional file 6: Table S5-Table S12 Gene content of type VI secretion system 1 (T6SS-1) found in all sequenced strains of *Pantoea ananatis*. This file contains all genes found within the contiguous region representing type VI secretion system 1 (PA T6SS-1) in all eight sequenced strains of *Pantoea ananatis* analysed in this study. For each strain of *P. ananatis,* the genes found in T6SS-1 are shown, including the product sizes, cluster of orthologous groups of proteins (COG) classification, conserved domain, subcellular localization, and the gene locus tags. Click here for file

Additional file 7: Table S13-Table S15 Gene content of type VI secretion system 2 (T6SS-2) in sequenced strains of *Pantoea ananatis*. This file contains all genes found within the contiguous region representing type VI secretion system 2 (PA T6SS-2) in different strains of *Pantoea ananatis*. For each strain of *P. ananatis*, the genes found in T6SS-2 are shown, including the product sizes, COG classification, conserved domain, subcellular localization, and the gene locus tags. Click here for file

Additional file 8: Figure S2 Alignment of the RhsD and RhsI proteins associated with *Pantoea ananatis* type VI secretion system 1. A) Shows alignment of the variable C-terminal domain of RhsD proteins from eight different strains *of P. ananatis*. This variable C-terminal domain is demarcated from the conserved N-terminal domain by a PxxxxxxDPxGL peptide motif indicated in the figure by blue stars. B) Shows alignment of the full length RhI proteins encoded by *rhsI* genes located downstream of the *rhsD* gene. Conserved residues are indicated by the different shadings. *P. ananatis* strains B1-9, LMG 2665^T^ and LMG 5342 have identical C-terminal extensions and encode identical RhsI homologs. Click here for file

Additional file 9: Figure S3 Alignment of all representative Hcp proteins encoded by strains of *Pantoea ananatis.* Representative Hcp amino acid sequences were aligned in BioEdit using ClustalW2 with default settings. The orphan Hcp proteins of *P. ananatis* strain LMG 20103 encoded by PANA_2446 and PANA_2447 are highly similar to the T6SS-associated Hcp protein encoded by PANA_2364. *P. ananatis* strain PA-13 encodes an additional orphan Hcp protein (PAGR_g3636) which is unique to this strain and is highly divergent from all other Hcp proteins. Click here for file

Additional file 10: Table S16 List of bacterial strains used for TssBC phylogenetic analysis. This file contains the list of TssB and TssC homologs from different bacterial species which were used for phylogenetic analysis. The accession numbers of all bacterial strains used in this study including the *tssB* and *tssD* gene locus tags are provided. Click here for file

Additional file 11: Table S17 List of bacterial strains used for Hcp phylogenetic analysis. This file contains all TssD homologs used for phylogenetic analysis. The accession numbers of all bacterial strains used in this study including the *tssD* gene locus tags are provided. Click here for file

Additional file 12: Figure S4 Hcp phylogenetic tree. The figure shows the evolutionary history of Hcp and “orphan” Hcp proteins of *Pantoea ananatis*. Phylogenetic analysis were conducted in MEGA6 [90]. The evolutionary history was inferred using the Maximum Likelihood method based on the General Reverse Transcriptase (rtRev) + I + G + F amino acid substitution models, as determined by ProtTest [91,97]. The percentage of replicate trees in which the associated taxa clustered together in the bootstrap test (1000 replicates) is shown next to the branches. The tree is drawn to scale, with branch lengths measured in the number of substitutions per site. Click here for file
