# Supplementary material for: Comparative genomics of type VI secretion systems in strains of Pantoea ananatis from different environments
Source: BMC Genomics. 2014 Feb 26;15:163. doi: 10.1186/1471-2164-15-163 (PMC3942780; doi:10.1186/1471-2164-15-163)
Supplement: Additional file 5: Figure S1 — Synteny between Pantoea ananatis type VI secretion system 1, 2 and 3 (PA T6SS-1, 2, 3). This file contains pairwise alignment of the homologous type VI secretion system gene clusters found in sequenced strains of P. ananatis. Alignments were generated using Mauve v.2.3.1 and show variable and conserved regions between the homologous T6SS gene clusters from different strains of P. ananatis. [file 1471-2164-15-163-S5.doc]

A: Alignment of the type VI secretion system-1 (T6SS-1) found in sequenced strains of *Pantoea ananatis*


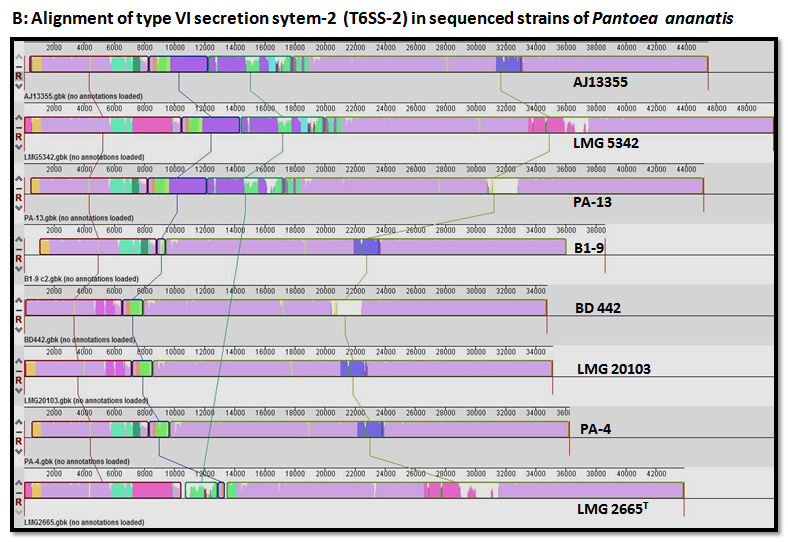


B: Alignment of type VI secretion system-2 (T6SS-2) found in sequenced strains of *Pantoea ananatis*


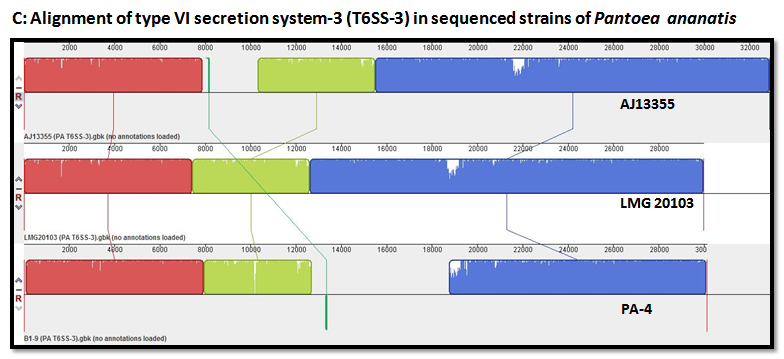


C: Alignment of the type VI secretion system-3 (T6SS-3) found in sequenced strains of *Pantoea ananatis*


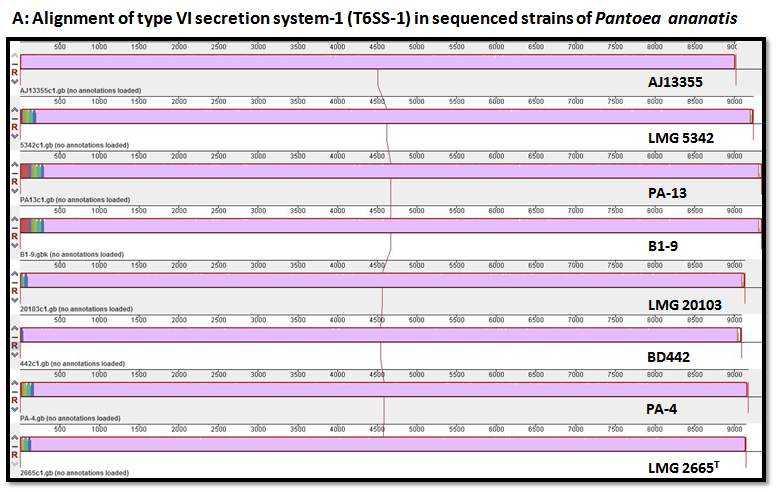


**Figure S1. Synteny between the different type VI secretion system clusters encoded by strains of *Pantoea ananatis***. T6SS-3 is highly syntenic in all strains analysed while PA T6SS-1 and PA T6SS-2 have variable regions and regions unique to each strain. These variable regions are usually clustered around *hcp* and *vgrG* genes and encode mostly hypothetical proteins.
