# Supplementary material for: Comparative genomics of type VI secretion systems in strains of Pantoea ananatis from different environments
Source: BMC Genomics. 2014 Feb 26;15:163. doi: 10.1186/1471-2164-15-163 (PMC3942780; doi:10.1186/1471-2164-15-163)
Supplement: Additional file 8: Figure S2 — Alignment of the RhsD and RhsI proteins associated with Pantoea ananatis type VI secretion system 1. A) Shows alignment of the variable C-terminal domain of RhsD proteins from eight different strains of P. ananatis. This variable C-terminal domain is demarcated from the conserved N-terminal domain by a PxxxxxxDPxGL peptide motif indicated in the figure by blue stars. B) Shows alignment of the full length RhI proteins encoded by rhsI genes located downstream of the rhsD gene. Conserved residues are indicated by the different shadings. P. ananatis strains B1-9, LMG 2665T and LMG 5342 have identical C-terminal extensions and encode identical RhsI homologs. [file 1471-2164-15-163-S8.docx]

**A**


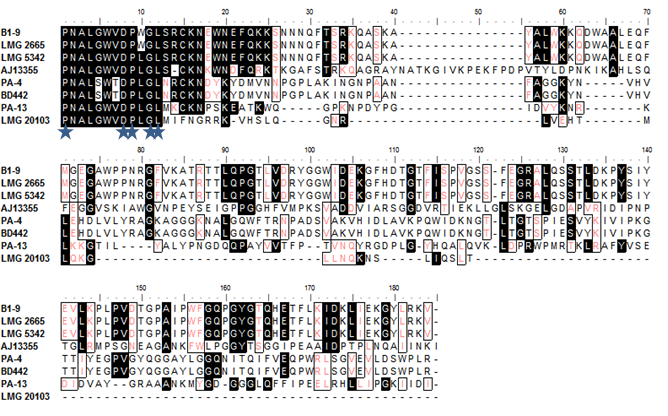


**B**

**Additional File 8. Alignment of the RhsD and RhsI proteins associated with *Pantoea ananatis* type VI secretion system 1**. A) Shows alignment of the variable C-terminal domain of RhsD proteins from eight different strains *of P. ananatis*. The variable C-terminal domain is demarcated from the conserved N-terminal domain by a PxxxxxxDPxGL peptide motif indicated in the figure by blue stars. B) Shows alignment of the full length RhI proteins encoded by *rhsI* genes located downstream of the *rhsD* gene. Conserved residues are indicated by the different shadings. *P. ananatis* strains B1-9, LMG 2665^T^ and LMG 5342 have identical C-terminal extensions and encoded identical RhsI homologs.
