# Supplementary material for: Comparative genomics of type VI secretion systems in strains of Pantoea ananatis from different environments
Source: BMC Genomics. 2014 Feb 26;15:163. doi: 10.1186/1471-2164-15-163 (PMC3942780; doi:10.1186/1471-2164-15-163)
Supplement: Additional file 9: Figure S3 — Alignment of all representative Hcp proteins encoded by strains of Pantoea ananatis. Representative Hcp amino acid sequences were aligned in BioEdit using ClustalW2 with default settings. The orphan Hcp proteins of P. ananatis strain LMG 20103 encoded by PANA_2446 and PANA_2447 are highly similar to the T6SS-associated Hcp protein encoded by PANA_2364. P. ananatis strain PA-13 encodes an additional orphan Hcp protein (PAGR_g3636) which is unique to this strain and is highly divergent from all other Hcp proteins. [file 1471-2164-15-163-S9.doc]

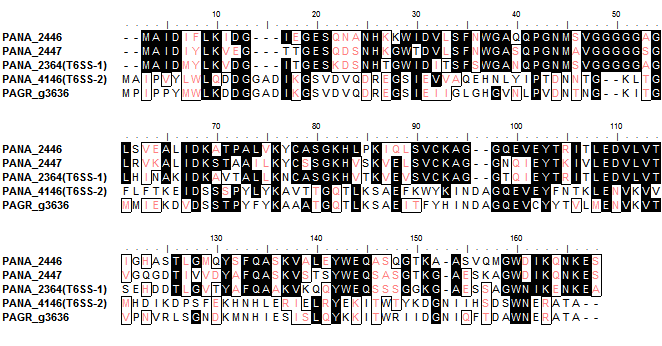


**Additional file 9. Alignment of all representative Hcp proteins encoded by strains of *Pantoea ananatis.*** Representative Hcp amino acid sequences were aligned in BioEdit using ClustalW2 with default settings. The orphan Hcp proteins of *P. ananatis* strain LMG 20103 encoded by PANA_2446 and PANA_2447 are highly similar to the T6SS-associated Hcp protein encoded by PANA_2364. *P. ananatis* strain PA-13 encodes an additional orphan Hcp protein (PAGR_g3636) which is unique to this strain and is highly divergent from all other Hcp proteins.
