# Supplementary material for: Comparative genomics of type VI secretion systems in strains of Pantoea ananatis from different environments
Source: BMC Genomics. 2014 Feb 26;15:163. doi: 10.1186/1471-2164-15-163 (PMC3942780; doi:10.1186/1471-2164-15-163)
Supplement: Supplementary file 10 [file 1471-2164-15-163-S12.doc]

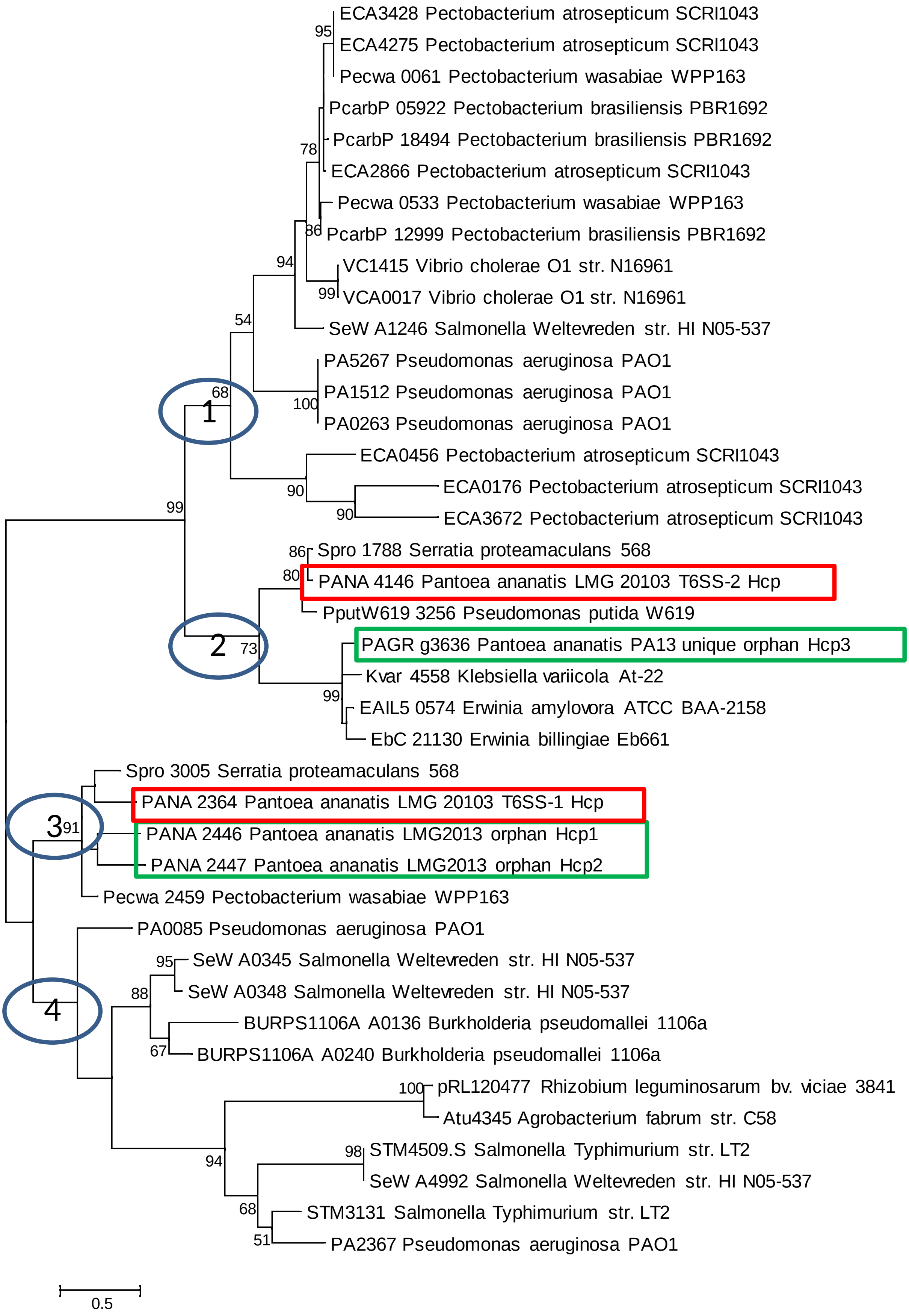

ECA3428 Pectobacterium atrosepticum SCRI1043
95
ECA4275 Pectobacterium atrosepticum SCRI1043
Pecwa 0061 Pectobacterium wasabiae WPP163
PcarbP 05922 Pectobacterium brasiliensis PBR1692
PcarbP 18494 Pectobacterium brasiliensis PBR1692
78
ECA2866 Pectobacterium atrosepticum SCRI1043
Pecwa 0533 Pectobacterium wasabiae WPP163
86
PcarbP 12999 Pectobacterium brasiliensis PBR1692
94
VC1415 Vibrio cholerae O1 str. N16961
99
VCA0017 Vibrio cholerae O1 str. N16961
54
SeW A1246 Salmonella Weltevreden str. HI N05-537
PA5267 Pseudomonas aeruginosa PAO1
68
PA1512 Pseudomonas aeruginosa PAO1
100
1
PA0263 Pseudomonas aeruginosa PAO1
ECA0456 Pectobacterium atrosepticum SCRI1043
90
ECA0176 Pectobacterium atrosepticum SCRI1043
99
90
ECA3672 Pectobacterium atrosepticum SCRI1043
86
Spro 1788 Serratia proteamaculans 568
80
PANA 4146 Pantoea ananatis LMG 20103 T6SS-2 Hcp
PputW619 3256 Pseudomonas putida W619
PAGR g3636 Pantoea ananatis PA13 unique orphan Hcp3
2
73
Kvar 4558 Klebsiella variicola At-22
99
EAIL5 0574 Erwinia amylovora ATCC BAA-2158
EbC 21130 Erwinia billingiae Eb661
Spro 3005 Serratia proteamaculans 568
PANA 2364 Pantoea ananatis LMG 20103 T6SS-1 Hcp
3
91
PANA 2446 Pantoea ananatis LMG2013 orphan Hcp1
PANA 2447 Pantoea ananatis LMG2013 orphan Hcp2
Pecwa 2459 Pectobacterium wasabiae WPP163
PA0085 Pseudomonas aeruginosa PAO1
95
SeW A0345 Salmonella Weltevreden str. HI N05-537
88
SeW A0348 Salmonella Weltevreden str. HI N05-537
4
BURPS1106A A0136 Burkholderia pseudomallei 1106a
67
BURPS1106A A0240 Burkholderia pseudomallei 1106a
100
pRL120477 Rhizobium leguminosarum bv. viciae 3841
Atu4345 Agrobacterium fabrum str. C58
98
STM4509.S Salmonella Typhimurium str. LT2
94
SeW A4992 Salmonella Weltevreden str. HI N05-537
68
STM3131 Salmonella Typhimurium str. LT2
51
PA2367 Pseudomonas aeruginosa PAO1
0.5
